# The deuterated glucose insulin tolerance test: a new tool to delineate insulin-stimulated glucose uptake from suppression of endogenous glucose production

**DOI:** 10.1093/lifemeta/loae036

**Published:** 2024-10-03

**Authors:** Christian A Unger, Marion C Hope, Michael Chase Kettering, Cassidy E Socia, Barton C Rice, Darya S Niamira, William E Cotham, Reilly T Enos

**Affiliations:** Department of Pathology, Microbiology, and Immunology, University of South Carolina-School of Medicine, Columbia, SC 29029, United States; Department of Pathology, Microbiology, and Immunology, University of South Carolina-School of Medicine, Columbia, SC 29029, United States; Department of Pathology, Microbiology, and Immunology, University of South Carolina-School of Medicine, Columbia, SC 29029, United States; Department of Pathology, Microbiology, and Immunology, University of South Carolina-School of Medicine, Columbia, SC 29029, United States; Department of Pathology, Microbiology, and Immunology, University of South Carolina-School of Medicine, Columbia, SC 29029, United States; Department of Pathology, Microbiology, and Immunology, University of South Carolina-School of Medicine, Columbia, SC 29029, United States; Department of Chemistry and Biochemistry, College of Arts and Science, University of South Carolina, Columbia, SC 29208, United States; Department of Pathology, Microbiology, and Immunology, University of South Carolina-School of Medicine, Columbia, SC 29029, United States

## Abstract

**Graphical Abstract:**
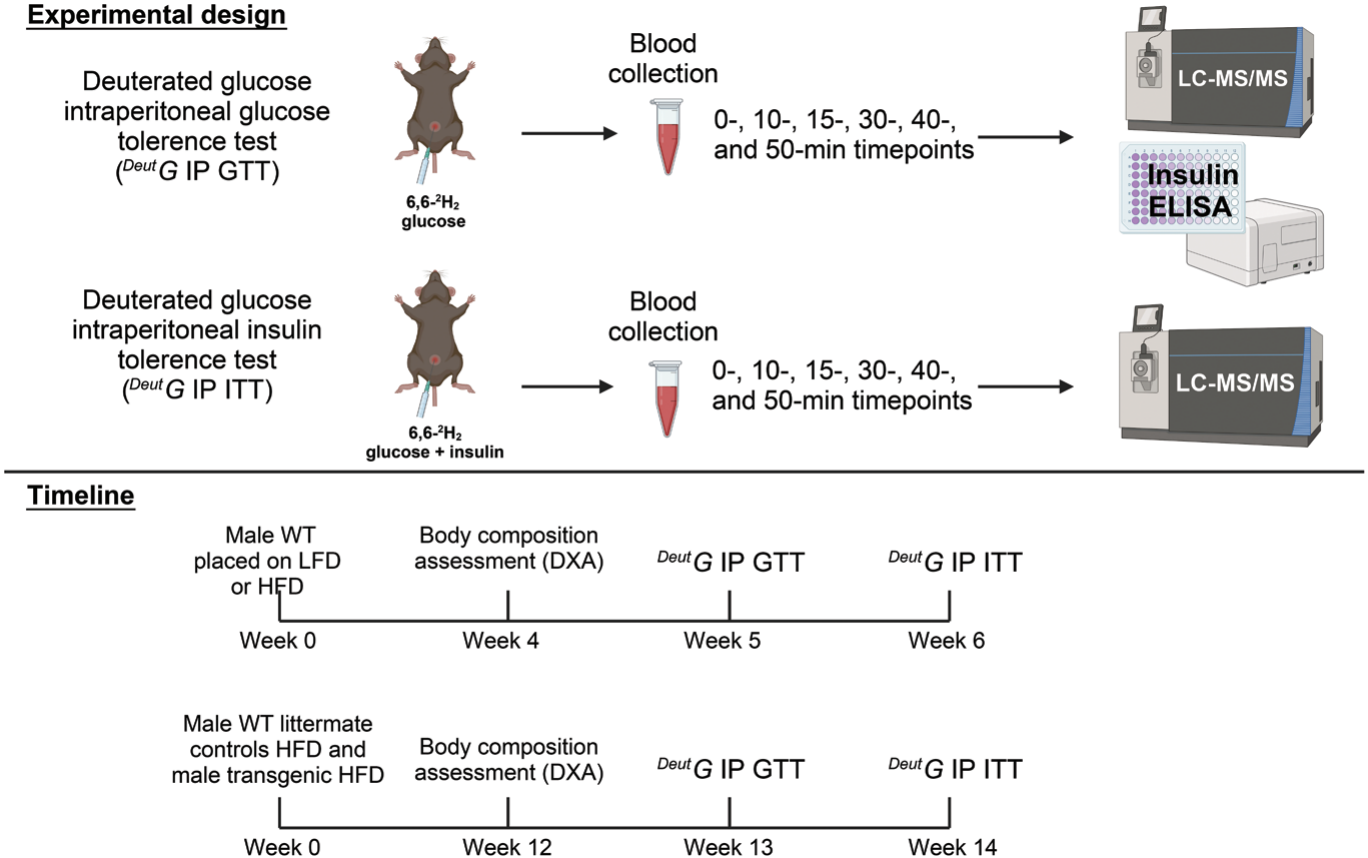

**Dear Editor**,

Assessing insulin responsiveness is crucial for understanding the underlying molecular mechanisms that contribute to disruptions in glucose metabolism. In preclinical research, glucose tolerance tests (GTTs) and insulin tolerance tests (ITTs) are widely regarded as essential tools due to their simplicity and affordability in assessing glucose homeostasis [1, 2]. Although these tests are excellent primary screeners for revealing dysregulated glucose metabolism, they are limited by their inability to ascertain any tissue-specific impairments to glucose metabolism. For instance, the ITT cannot differentiate between deficiencies in insulin-stimulated glucose uptake and insulin’s effectiveness in suppressing endogenous glucose production (EGP) (hepatic insulin sensitivity). Therefore, while GTTs and ITTs are valuable for their accessibility and ease of implementation, more advanced techniques such as the hyperinsulinemic-euglycemic clamp are necessary for investigating tissue-specific aspects of insulin responsiveness.

The hyperinsulinemic-euglycemic clamp is considered as the gold standard for assessing insulin sensitivity, as it allows for the evaluation of both hepatic insulin sensitivity, when radiolabeled tracers are used, and glucose disposal [3]. In addition, when radiolabeled 2-deoxyglucose is incorporated into the experiment, tissue-specific glucose uptake can be determined [3]. Despite its accuracy, the clamp requires skilled surgical expertise for venous (at the minimum) and arterial catheterization, involves a substantial time commitment, uses radiolabeled isotopes, and is relatively expensive. Moreover, the clamp does not assess basal glucose uptake. These limitations are part of the rationale for the recent development of the dual-tracer test, which allows for the assessment of tissue-specific basal and insulin-stimulated glucose transport using two radiolabeled tracers [4]. Although the dual-tracer test is effective in assessing tissue-specific glucose uptake, is much easier to perform than the hyperinsulinemic-euglycemic clamp, and is more cost-effective, a limitation of this test is its inability to assess EGP suppression.

Given the shortcomings of current tests used by preclinical metabolic researchers, our goal was to create a straightforward, sensitive, and reliable metabolic assessment method that would enable simultaneous determination of insulin-stimulated glucose uptake and insulin-induced suppression of EGP. Through this approach, we demonstrate the capability to not only sensitively detect deficiencies in insulin-stimulated exogenous glucose disposal but also identify subtle impairments in insulin-induced EGP suppression even without the presence of hyperinsulinemia.

Details regarding animal use and methodology are provided in Supplementary Materials and Methods. In brief, male mice on C57BL/6J background were used for all experiments. At 12 weeks of age, mice were assigned to receive either a purified low-fat diet (LFD) (*n* = 14) or a custom high-fat diet (HFD) (*n *= 15) [5]. To illustrate the practical application of the metabolic tests discussed in this manuscript, we have included previously unpublished data from a long-term HFD study involving an undisclosed transgenic mouse model and wild-type (WT) littermates. For this “transgenic” experiment, male mice on a C57BL/6 background consumed the HFD for 13 weeks prior to metabolic assessment. The experimental designs of these studies are presented in the Graphical Abstract. Body composition was assessed after four weeks of diet (12 weeks of HFD for the transgenic experiment) to use lean mass as the basis for the dose of glucose and insulin administration for subsequent metabolic tests.

The appropriate protocol for the assessment of insulin-stimulated glucose uptake and suppression of EGP involves two metabolic tests: the deuterated glucose intraperitoneal GTT (^*Deut*^*G* IP GTT) and the ^*Deut*^*G* IP ITT. The ^*Deut*^*G* IP GTT, otherwise known as a stable-isotope labeled GTT described elsewhere [6–8], involves the administration of deuterated glucose ([6-6-^2^H_2_] glucose), a stable isotope of glucose, in the place of non-isotopically labeled glucose. As previously determined, intraperitoneally (IP) administered [6-6-^2^H_2_] glucose does not significantly recirculate into the blood once taken up by the tissue [8]. Therefore, by monitoring blood glucose concentrations and collecting blood samples throughout the metabolic test, one can determine exogenous glucose disposal by assessing [6-6-^2^H_2_] glucose in circulation. In addition, by determining the ratio of [6-6-^2^H_2_] glucose to unlabeled glucose, one can differentiate between exogenous glucose uptake and EGP. The advantage of IP administered glucose is that it does not cause a spike in blood insulin levels in contrast to oral administration of glucose [6]. As such, one can determine “insulin-independent” glucose uptake, otherwise known as “glucose handling” [1], and the “insulin-independent” effect of exogenous glucose administration on EGP.

The *^Deut^G* IP ITT differs from the ^*Deut*^*G* IP GTT in that insulin and [6-6-^2^H_2_] glucose are administered simultaneously. This allows for the determination of insulin-stimulated glucose uptake as well as the ability of insulin to inhibit EGP. Pairing the results from the ^*Deut*^*G* IP GTT and ^*Deut*^*G* IP ITT helps to tease out whether the phenotypes exhibited are primarily a result of differences in “glucose handling” and/or insulin action.

The ^*Deut*^*G* IP GTT and ^*Deut*^*G* IP ITT were performed on the same animals, with a 1-week separation between the two tests. For these tests, the mice were fasted for 3 h [9] and were IP injected with [6-6-^2^H_2_] glucose (Cambridge Isotope Laboratories, Tewksbury, MA) with or without insulin (Sigma Aldrich, St. Louis, MO). For the ^*Deut*^*G* IP ITT, [6-6-^2^H_2_] glucose and insulin were provided in the same insulin syringe. The doses of glucose and insulin administered were normalized to the lean body mass of the animal—0.5 g/kg lean mass and 0.75 U/kg lean mass, respectively. A total volume of 150 µL was administered to each animal, which was brought up to equal volume with the addition of physiological saline. A glucometer (Bayer Contour, Mishawaka, IN) was used to measure blood glucose concentrations (tail sampling) intermittently over a 50-min period (0, 10, 15, 30, 40, and 50 min). During the same period, blood was collected from the tip of the tail via heparinized capillary tubes to differentiate isotopically labeled glucose from endogenous glucose using LC-MS/MS (detailed in Supplementary Materials and Methods) and to determine blood insulin concentrations (^*Deut*^*G* IP GTT only) via enzyme-linked immunosorbent assay (ELISA) (Mercodia, Uppsala, Sweden). The area of the curve (AOC) after subtracting baseline blood glucose concentrations was calculated using the trapezoidal rule based on the insights by Virtue and Vial-Puig [1].

As expected, HFD consumption elicited a greater increase in body weight gain than LFD consumption (Fig. 1a). However, both the LFD and HFD-fed mice presented similar lean mass contents (Fig. 1b). Therefore, both the LFD and HFD-fed mice received similar amounts of glucose and/or insulin in subsequent metabolic tests. Five weeks of HFD consumption was sufficient to induce hyperglycemia (Fig. 1c), but not hyperinsulinemia (Fig. 1d) (*P* < 0.05). Furthermore, we confirmed that the ^*Deut*^*G* IP GTT did not induce changes in blood insulin levels (Fig. 1d), allowing us to determine that five weeks of HFD consumption was sufficient to impair glucose handling independent of changes to circulating insulin (Fig. 1c, d, and f).

**Figure 1:**
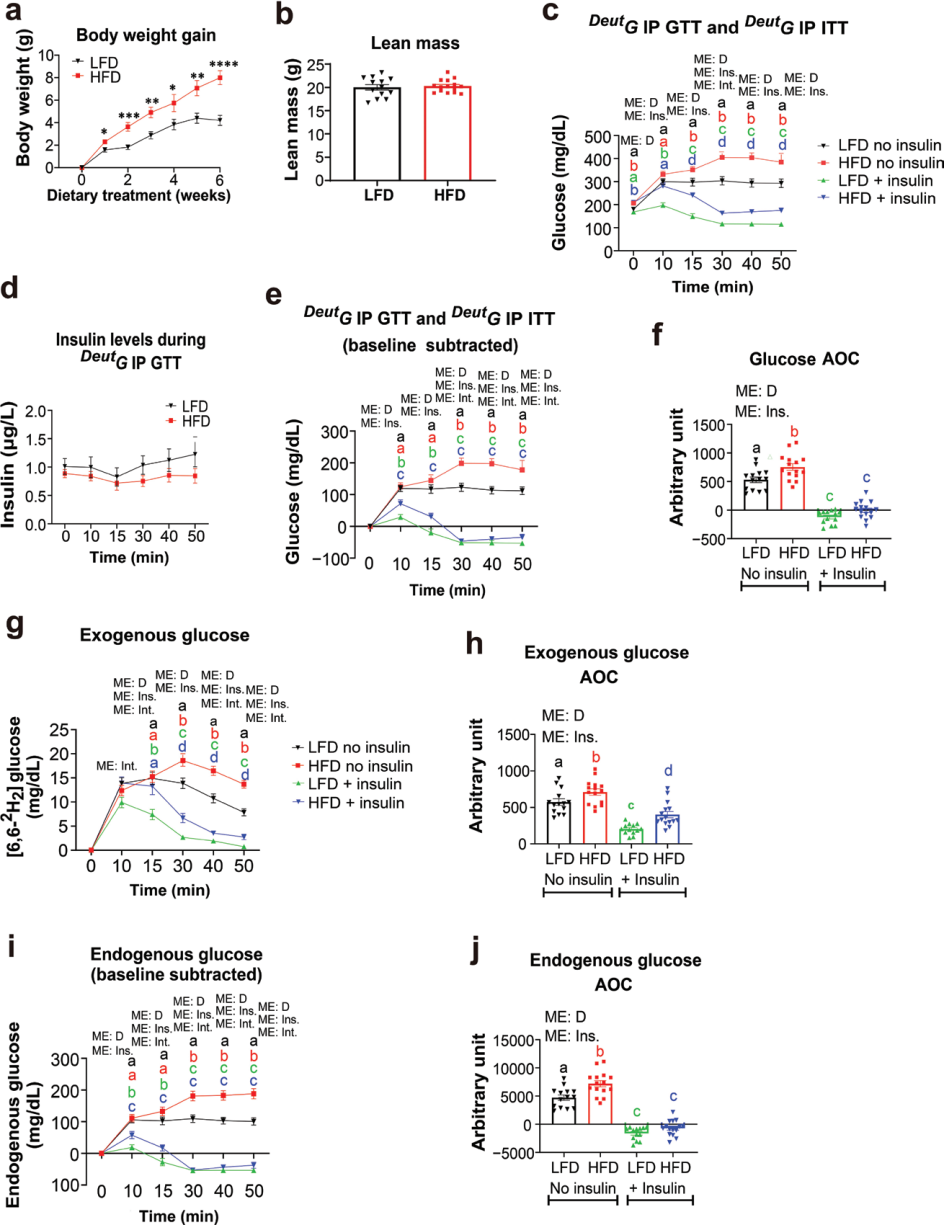
The ^*Deut*^*G* IP GTT and ^*Deut*^*G* IP ITT provide insights into both endogenous and exogenous glucose metabolism. Male mice on a C57BL/6 background were fed with either a LFD (*n *= 14) or an HFD (*n* = 15) for six weeks. (a) Body weight gain throughout the dietary treatment. (b) Lean mass assessment via dual-energy X-ray absorptiometry (DXA). (c) Raw glucose data for the ^*Deut*^G IP GTT and ^*Deut*^G IP ITT. (d) Plasma insulin levels during the ^*Deut*^*G* IP GTT. (e) Glucose levels throughout the ^*Deut*^*G* IP GTT and ^*Deut*^*G* IP ITT accounting for baseline glucose levels. (f) AOC of the baseline glucose subtracted ^*Deut*^*G* IP GTT and ^*Deut*^*G* IP ITT. (g) Blood [6-6-^2^H_2_] glucose concentrations throughout the ^*Deut*^*G* IP GTT and ^*Deut*^*G* IP ITT representing exogenous glucose. (h) AOC of exogenous glucose. (i) Endogenous blood glucose concentrations throughout the ^*Deut*^*G* IP GTT and ^*Deut*^*G* IP ITT. (j) AOC of endogenous glucose. Data are presented as means ± SE. ^*^, ^**^, ^***^, and ^****^ signifies *P* < 0.05, < 0.01, < 0.001, and < 0.0001, respectively. Bar graphs or *x*-*y* plots not sharing a common letter are significantly different from one another (*P* < 0.05). ME = main effect; D = diet; Ins. = insulin; Int. = interaction.

One week after the ^*Deut*^*G* IP GTT, the ^*Deut*^*G* IP ITT was performed. It was evident that despite impairments to glucose handling, the HFD-fed mice were still responsive to insulin as determined by the AOC (Fig. 1f) (*P* < 0.05). Nonetheless, the LFD-fed mice, when treated with insulin, were found to be more responsive to insulin at the 10- and 15-min timepoints than the HFD-fed mice (Fig. 1e) (*P* < 0.05). When exogenous glucose was discriminated from endogenous glucose, it was evident that LFD-fed mice displayed enhanced exogenous glucose handling (Fig. 1g and h) (*P* < 0.05). In addition, when administered insulin, despite no difference in the AOC, the LFD-fed mice presented superior exogenous glucose uptake, which was apparent starting at the 15-min timepoint (Fig. 1f and g) (*P* < 0.05). It should also be noted that by performing both the ^*Deut*^*G* IP GTT and ^*Deut*^*G* IP ITT, we were able to determine that the HFD-fed mice were still responsive to insulin with respect to exogenous glucose disposal (Fig. 1f and g) (*P* < 0.05). What is important to point out is that the ^*Deut*^*G* IP ITT was able to determine impairments in exogenous glucose disposal (Fig. 1g) that were not able to be uncovered by examining non-isotope discriminated glucose concentrations alone (Fig. 1e). In fact, relying solely on non-isotope discriminated glucose concentrations (Fig. 1e) could lead to the incorrect conclusion that there is no difference in insulin-stimulated glucose uptake between treatment groups. However, the sensitivity of the ^*Deut*^*G* IP ITT clearly distinguished the difference in insulin-stimulated glucose uptake between the groups (Fig. 1g).

With respect to endogenous glucose metabolism, it was evident that during the ^*Deut*^*G* IP GTT, the HFD-fed mice presented augmented EGP relative to LFD-fed mice (Fig. 1i and j) (*P* < 0.05). However, upon insulin administration, the HFD-fed mice displayed suppressed EGP to a similar extent as the LFD-fed mice (Fig. 1i and j) (*P* < 0.05). However, even though the endogenous glucose AOC was similar between the groups, the LFD-fed mice suppressed EGP more rapidly (10- and 15-min timepoints) than the HFD-fed mice (Fig. 1i) (*P* < 0.05). A key observation from these tests is that the endogenous glucose graphs presented in Fig. 1i and j closely mimic the undifferentiated glucose isotope graphs presented in Fig. 1e and f, respectively. This suggests that a conventional ITT, due to its lack of sensitivity, primarily assesses endogenous glucose metabolism rather than glucose uptake by tissues. This is likely due to the significant amount of endogenous glucose produced and released into circulation, which exceeds the amount of glucose taken up by tissues in response to a single bolus of insulin. Therefore, insulin’s suppression of EGP has a more pronounced impact on circulating glucose levels compared to its stimulation of glucose uptake. This suggests that unless a sensitive test like the ^*Deut*^*G* IP GTT or a test in which radioisotopes are utilized, it may not be possible to assess impairments in glucose uptake with a traditional ITT. Rather a traditional ITT in most cases would likely only determine impairments with respect to insulin-induced EGP suppression.

To provide an additional preclinical example of how the ^*Deut*^*G* IP ITT may be used experimentally, we included unpublished data from our laboratory. In this example, we fed male transgenic mice and WT littermates with 40% HFD for 12 weeks followed by body composition analysis and subsequent ^*Deut*^*G* IP GTT and ^*Deut*^*G* IP ITT metabolic tests. Both WT and transgenic mice presented similar body weight gain and lean body mass (Supplementary Fig. S1a and b). Even though there were no differences with respect to glucose handling, the transgenic mice presented enhanced insulin action relative to WT mice (Supplementary Fig. S1c−e) (*P* < 0.05). When parsing out exogenous versus endogenous glucose metabolism, it was apparent, despite no difference in the AOC, that the transgenic mice had superior insulin-stimulated glucose disposal as evidenced by lower exogenous glucose levels at 30, 40, and 50 min relative to WT mice (Supplementary Fig. S1f and g) (*P* < 0.05). Furthermore, with respect to insulin-stimulated EGP suppression, the transgenic mice displayed enhanced EGP suppression relative to WT mice (Supplementary Fig. S1h and i) (*P* < 0.05). It was of interest to observe that after 13−14 weeks of HFD consumption, WT mice responded to insulin with respect to exogenous glucose disposal, but this was not the case for EGP suppression (Supplementary Fig. S1g and i). Furthermore, this second experiment reinforces the notion that a conventional ITT primarily reflects endogenous glucose metabolism. This is underscored by the resemblance in glucose patterns observed in Supplementary Fig. S1d and h. Notably, Supplementary Fig. S1f illustrates subtle differences in exogenous glucose uptake, which would not be discernible with a less sensitive test.

Besides its role in enhancing post-prandial glucose uptake, insulin is a potent regulator of endogenous glucose metabolism primarily through its suppressive action on hepatic EGP [10, 11]. Impairments in hepatic insulin sensitivity have been shown to precede skeletal muscle and adipose tissue insulin resistance in HFD-fed mouse and rat models [12–14]. Therefore, the ability to assess both insulin’s impact on glucose uptake and suppression of EGP is of great interest as it would provide insights to the metabolic researchers to narrow down the tissue and potential mode of action of a nutritional intervention, drug treatment, or genetically modified mouse model resulting in altered insulin sensitivity.

Currently, preclinical metabolic researchers primarily rely on the ITT to assess alterations to insulin action. However, this test is limited in several aspects. For example, insulin-sensitive animals with lower fasting blood glucose levels will undergo hypoglycemia which will elicit a counterregulatory survival response that may lead to an underestimation of insulin sensitivity [2, 4]. In addition, the ITT does not distinguish whether the drop in blood glucose resulting from insulin administration is due to glucose uptake and/or suppression of EGP. Currently, for metabolic researchers to delineate whether the effects of insulin are due to changes in glucose disposal, a suppression of EGP, or both, a hyperinsulinemic-euglycemic clamp must be performed. Unfortunately, most researchers are not able to perform this specialized experiment for a variety of reasons, including monetary cost, the need for a skilled surgeon, and the time required to perform the procedure with an adequate sample size. For these reasons, we developed the ^*Deut*^*G* IP ITT and have highlighted the distinctions among frequently utilized metabolic tests in preclinical research in Table 1.

**Table 1:** Summary of metabolic tests commonly used in preclinical metabolic research to assess insulin sensitivity.

Test	Expense	Surgical requirement?	Determining differences in tissue-specific glucose uptake?	Distinguishing endogenous and exogenous glucose metabolism?	Anesthesia requirement?	Assessing basal glucose uptake?	Use of radiolabeled isotopes?
Insulin tolerance test	$	No	No	No	No	No	No
Hyperinsulinemic-euglyemic clamp	$$$	Yes	No^a^	Yes	No	No	Yes, typically
Dual-tracer test	$$	No	Yes	No	Yes	Yes	Yes
^*Deut*^ *G* IP ITT	$$	No	No^a^	Yes	No	No	No

^a^Unless used in conjunction with radiolabeled 2-deoxyglucose.

The ^*Deut*^*G* IP ITT takes advantage of the use of a deuterated glucose isotope to be able to differentiate between exogenous and endogenous glucose. While a deuterated glucose isotope has been used when performing GTTs [6–8, 15], as far as we are aware, it has not been used in the setting of assessing insulin action. The value of the ^*Deut*^*G* IP ITT is that it assesses the effect of insulin on exogenous glucose disposal as well as suppression of EGP. When developing the test, we concluded that, ideally, a ^*Deut*^*G* IP GTT would be performed in addition to the ^*Deut*^*G* IP ITT. This approach is rationalized by the fact that the ^*Deut*^*G* IP ITT involves glucose administration and glucose handling/effectiveness significantly influences glucose disposal independent of changes to insulin levels [6, 15]. We deliberately opted for a relatively modest dose of deuterated glucose (0.5 mg/kg lean mass) and confirmed that this dose does not affect insulin levels. Therefore, conducting both the ^*Deut*^*G* IP GTT and ^*Deut*^*G* IP ITT allows researchers to discern whether observed effects stem from genuine insulin action, glucose handling capabilities, or a combination of both. Moreover, by administering glucose during the test, we prevented the severe hypoglycemia often induced in highly insulin-sensitive animals during the conventional ITT. This circumvention of severe hypoglycemia enhances the accuracy of the metabolic assessment and ensures more reliable data interpretation. In addition, since neither surgery nor radioisotopes are required, the ^*Deut*^*G* IP GTT and ^*Deut*^*G* IP ITT can be employed to evaluate insulin action in longitudinal and time-course studies and can be applied to other preclinical models, such as rats and larger animals, although this will incur an additional cost due to the need for more deuterated glucose to accommodate the increased body mass.

Our results highlight the sensitivity and the power of the ^*Deut*^*G* IP ITT, as we successfully identified variations in exogenous glucose disposal that would have been overlooked when analyzing undifferentiated, “raw” glucose levels alone. Furthermore, our data indicate that the conventional ITT, owing to its lower sensitivity, primarily evaluates insulin’s capacity to suppress EGP rather than exogenous glucose disposal. This is an important fact for researchers to be aware of when selecting metabolic tests to appropriately tease out a given metabolic phenotype.

It is important to acknowledge the limitations of the ^*Deut*^*G* IP ITT in comparison to the traditional ITT. The main constraint is the necessity for a mass spectrometer capable of distinguishing between endogenous and exogenous glucose. Moreover, there is a higher cost associated with the ^*Deut*^*G* IP ITT compared to a standard ITT, encompassing expenses such as the per-sample run cost on a mass spectrometer and the price of the deuterated glucose. Despite these costs, the test offers superior sensitivity and ability to differentiate between the effects of insulin on exogenous and endogenous glucose metabolism, a distinction previously only achievable with a hyperinsulinemic-euglycemic clamp. Moreover, the ^*Deut*^G IP ITT has been proved to be more cost-effective than the latter, making it a valuable tool for metabolic researchers aiming to probe insulin responsiveness in greater detail without the additional cost required for a hyperinsulinemic-euglycemic clamp or the need for radiolabeled isotopes. Nevertheless, it is important to note that in an ideal scenario, the hyperinsulinemic-euglycemic clamp would still be preferred due to its unmatched sensitivity in assessing insulin sensitivity.

In conclusion, the ^*Deut*^G IP ITT represents an advancement in metabolic research, offering a nuanced assessment of insulin action on both exogenous glucose disposal and EGP suppression. The test provides insights that a traditional ITT cannot match, highlighting the sensitivity of the ^*Deut*^G IP GTT. While acknowledging the need for specialized equipment and higher initial costs, the ^*Deut*^G IP ITT is proved to be a more accessible alternative to the hyperinsulinemic-euglycemic clamp, which is often impractical for many researchers due to logistical or financial constraints. By enabling researchers to dissect insulin’s actions more comprehensively and accurately, the ^*Deut*^G IP ITT stands poised to enhance our understanding of metabolic phenotypes and facilitate the evaluation of interventions targeting insulin sensitivity in diverse experimental settings.

## Supplementary Material

loae036_suppl_Supplementary_Material

## Data Availability

The authors confirm that all the data supporting the findings of this study are available within the supplementary material and corresponding authors.
