# Supplementary material for: The deuterated glucose insulin tolerance test: a new tool to delineate insulin-stimulated glucose uptake from suppression of endogenous glucose production
Source: Life Metab. 2024 Oct 3;4(1):loae036. doi: 10.1093/lifemeta/loae036 (PMC11770813; doi:10.1093/lifemeta/loae036)
Supplement: loae036_suppl_Supplementary_Material [file loae036_suppl_supplementary_material.pdf]

Supplementary materials and methods

Animals and diets

Male mice on C57BL/6J background were used for all experiments. At 12 weeks of age, mice were assigned to receive either a purified low-fat diet (LFD) (*n* = 14) or a custom high-fat diet (HFD) (*n* = 15). The LFD utilized in this experiment was the open-source, purified AIN-76A diet (3.77 kcal/g). The HFD (4.57 kcal/g) was a purified diet comprised of 47%, 40%, and 13% of total calories from carbohydrate, fat, and protein, respectively, with saturated fat making up 12% of total calories to mimic the standard American diet (BioServ, Frenchtown, NJ). Details and previous use of this diet are provided elsewhere[1-9]. To illustrate the practical application of the metabolic tests discussed in this manuscript, we have included previously unpublished data from a long-term HFD study involving an undisclosed transgenic mouse model and wild-type (WT) littermates. For this "transgenic" experiment, male mice on a C57BL/6 background consumed the HFD for 13 weeks before metabolic assessment. The experimental designs of these studies are presented in the Graphical Abstract. Mice were housed, 4–5/cage, maintained on a 12-h light/12-h dark cycle in a low stress environment (22.5°C, 50% humidity, low noise), and given food and water *ad libitum*. All methods were in accordance with the American Association for Laboratory Animal Science, and the Institutional Animal Care and Usage Committee of the University of South Carolina approved all experiments.

Body weight and body composition

Body weight was assessed weekly throughout the dietary treatment. Body composition was assessed after four weeks of diet (12 weeks of HFD for the transgenic experiment) to use lean mass as the basis for the dose of glucose and insulin administration for subsequent metabolic tests. For this procedure, mice were briefly anesthetized via isoflurane inhalation, and body composition was assessed by dual-energy X-ray absorptiometry (DXA) (Lunar PIXImus, Fitchburg WI).

Chromatography and LC-MS/MS for glucose and [6-6-$^2H_2$] glucose assessment

A total of 180 µL 80% mass-spec grade methanol (Fisher Scientific, Waltham, MA) was added to 2 µL plasma to extract glucose for LC-MS/MS analysis (Fisher Scientific, Waltham, MA) as previously described[10]. Analyses were carried out on a Q Exactive HF-X hybrid quadrupole-orbitrap mass spectrometer with a Vanquish HPLC on the front end (Thermo Electron, Waltham, MA). The sample (2 µL) was injected onto a Waters Xbridge Amide column (2.1 mm × 100 mm, 3.5-µm particles; Waters Corp. Milford, MA) using a mobile phase consisting of 0.1% ammonium hydroxide in 80% acetonitrile, 20% water (A) and 0.1% ammonium hydroxide in 30% acetonitrile, 70% water (B). Glucose and [6-6-$^2H_2$] glucose were analyzed using the following linear solvent gradient at a flow rate of 250 µL/min: 0% B at initial conditions ramping to 12% B over 6 min followed by a sharp ramp to 50% B ending at 7 min. At 7.1 min, a return to starting conditions of 0% B ending at 10 min. Mass analysis was carried out using electrospray ionization in the negative ion mode. The HESI electrospray ionization source settings were as follows: Sheath gas flow, 35 units; Aux gas flow, 10 units; Sweep gas flow, 2 units; Electrospray voltage, 2.8 kV; Capillary temperature, 275 °C; Funnel RF, 40; Aux gas heater, 300 °C. The glucose and [6-6-$^2H_2$] glucose were analyzed in MSMS mode using parallel reaction monitoring (PRM). The precursor ion for glucose was the M-H ion at *m/z* 179.0561; the precursor ion for [6-6-$^2H_2$] glucose was the M-H at *m/z* 181.0687. The precursor isolation window was 1.0 *m/z*. A normalized collision energy (NCE) of 10 V was used for both analytes. PRM settings on the QE-HF-X were as follows: negative ion polarity; default charge state of 1; microscans = 1; mass resolution set to 120,000; AGC target of 2E5; and maximum IT of 200 ms. For quantitation the [M-$C_3H_6O_3$] product ion from each analyte was monitored (for glucose *m/z* 89.0244 and for [6-6-$^2H_2$] glucose *m/z* 91.0370). The high resolution of 120,000 prevented the natural glucose isotopomers from interfering with the measurement of the [6-6-$^2H_2$] glucose production. The mass spectrometer was mass calibrated before each batch of runs and the data was collected and processed using the vendor-provided Xcalibur software with mass error filter set at 5 ppm. The fractional abundance of [6-6-$^2H_2$] glucose and

unlabeled glucose was calculated and multiplied by the total glucose determined by the glucometer to calculate the absolute amounts of exogenous and endogenous glucose.

Statistical analysis

Graphpad Prism Software (La Jolla, CA) was used for all statistical analyses. A Student's Two-Tailed *t*-test was used for assessing differences in weekly body weight changes throughout the dietary treatment as well as for lean body mass assessment. Blood insulin concentrations throughout the ${}^{Deut}G$ IP GTT between LFD and HFD-fed mice were assessed using a repeated-measured Two-Way ANOVA with a Student Newman-Keuls *post hoc* test to determine whether there were any changes in blood insulin levels from baseline values. A Two-Way ANOVA (accounting for diet and insulin administration or genotype and insulin administration for the transgenic animal experiment) with a Student Newman-Keuls *post hoc* test was used for all other statistical comparisons. Any statistical test that did not pass the equal-variance test (Bartlett's test for equal variances) was log- or cube-root-transformed and reanalyzed. Data are presented as means ± SE, and the level of significance was set at $P < 0.05$.

REFERENCES

1. Enos RT, Velazquez KT, McClellan JL *et al*. Lowering the dietary omega-6: omega-3 does not hinder nonalcoholic fatty-liver disease development in a murine model. *Nutr Res* 2015;**35**: 449−59.
2. Enos RT, Velazquez KT, Murphy EA. Insight into the impact of dietary saturated fat on tissue-specific cellular processes underlying obesity-related diseases. *J Nutr Biochem* 2014;**25**: 600−12
3. Enos RT, Velazquez KT, McClellan JL *et al.* High-fat diets rich in saturated fat protect against azoxymethane/dextran sulfate sodium-induced colon cancer. *Am J Physiol Gastrointest Liver Physiol* 2016;**310**:G906−19.
4. Velazquez KT, Enos RT, Carson MS *et al*. miR155 deficiency aggravates high-fat diet-induced adipose tissue fibrosis in male mice. *Physiol Rep* 2017;**5**:e13412.
5. Bader JE, Enos RT, Velazquez KT *et al.* Repeated clodronate-liposome treatment results in neutrophilia and is not effective in limiting obesity-linked metabolic impairments. *Am J Physiol Endocrinol Metab* 2019;**316**:E358−72.

6. Cranford TL, Enos RT, Velazquez KT *et al*. Role of MCP-1 on inflammatory processes and metabolic dysfunction following high-fat feedings in the FVB/N strain. *Int J Obes (Lond)* 2016;**40**:844−51.
7. Enos RT, Davis JM, Velazquez KT *et al*. Influence of dietary saturated fat content on adiposity, macrophage behavior, inflammation, and metabolism: composition matters. *J Lipid Res* 2013;**54**:152−63.
8. Enos RT, Velazquez KT, Carson MS *et al*. A Low Dose of Dietary Quercetin Fails to Protect against the Development of an Obese Phenotype in Mice. *PLoS One* 2016;**11**:e0167979.
9. Aladhami AK, Unger CA, Ennis SL *et al*. Macrophage tumor necrosis factor-alpha deletion does not protect against obesity-associated metabolic dysfunction. *FASEB J* 2021;**35**:e21665.
10. Zhang Y, Xu L, Liu X *et al*. Evaluation of insulin sensitivity by hyperinsulinemic-euglycemic clamps using stable isotope-labeled glucose. *Cell Discov* 2018;**4**:17.

Supplementary Figure S1

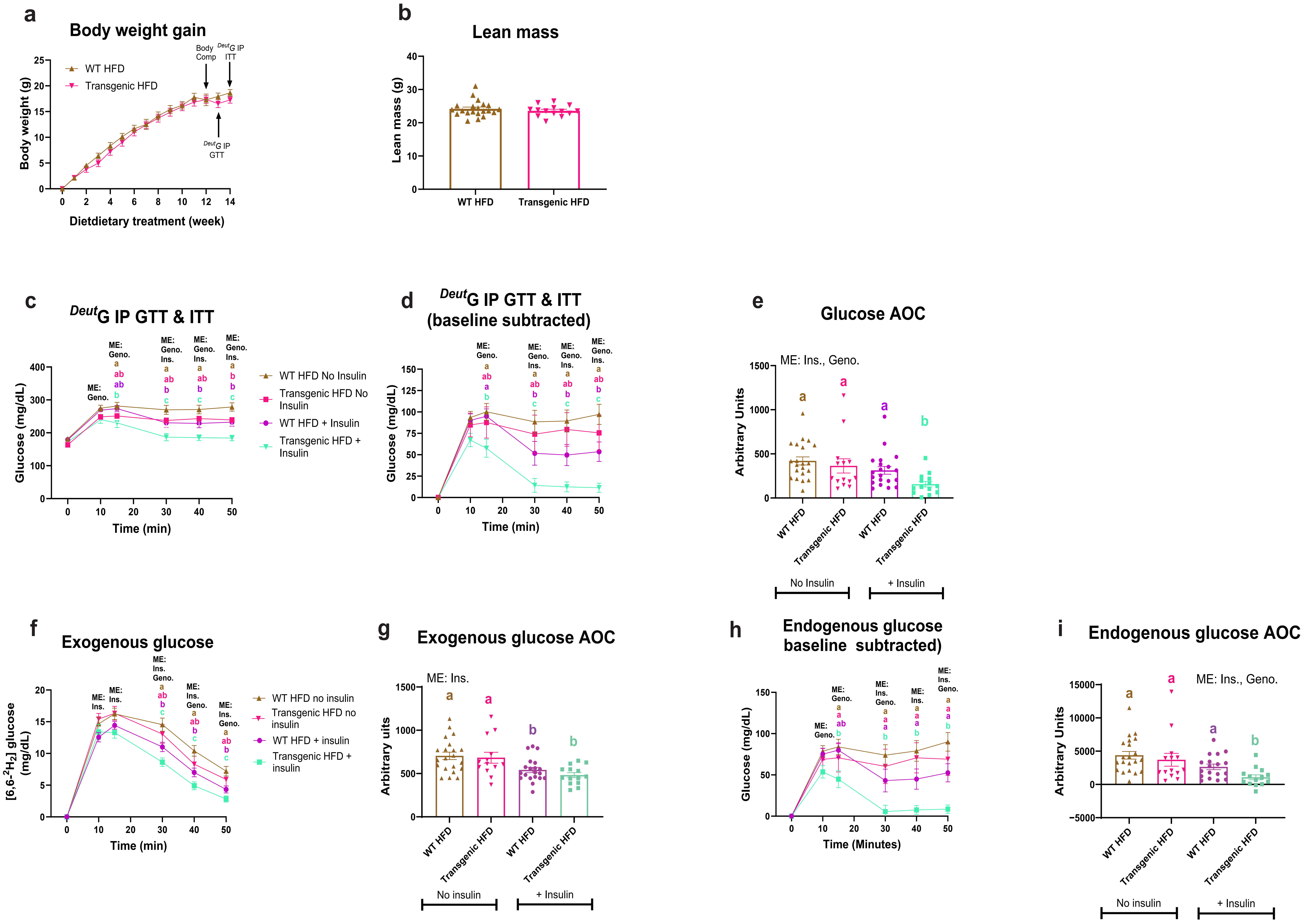

Supplementary Figure S1 The ^{Deut}G IP GTT and ^{Deut}G IP ITT have the capability to tease out differences with respect to insulin action independent of changes to glucose handling. Male wild-type (WT) littermates (n = 21) and transgenic mice (n = 13) on C57BL/6 background were fed with an HFD for 14 weeks. (a) Body weight gain throughout the dietary treatment. (b) Lean mass assessment via DXA. (c) Raw glucose data for the ^{Deut}G IP GTT and ^{Deut}G IP ITT. (d) Glucose levels throughout the ^{Deut}G IP GTT and ^{Deut}G ITT accounting for baseline glucose levels. (e) Area of the curve (AOC) of the baseline glucose subtracted ^{Deut}G IP GTT and ^{Deut}G IP ITT. (f) Blood [6-6-$^{2}H_2$] glucose concentrations throughout the ^{Deut}G IP GTT and ^{Deut}G IP ITT representing exogenous glucose. (g) Exogenous glucose AOC. (h) Endogenous blood glucose concentrations over the course of the ^{Deut}G IP GTT and ^{Deut}G IP ITT. (j) Endogenous glucose AOC. Data are presented as means ± SE. Bar graphs or x-y plots not sharing a common letter are significantly different from one another (p <0.05). ME = main effect; Ins. = insulin; Geno. = genotype.
